# HPV vaccination coverage in the federal state of Belgium according to regions and their impact

**Published:** 2018-06

**Authors:** WAA Tjalma, C Brasseur, G Top, N Ribesse, I Morales, PA Van Damme

**Affiliations:** Multidisciplinary Breast cancer clinic, Gynaecological Oncology Unit, Department of Obstetrics & Gynaecology, Antwerp University Hospital, University of Antwerp, Wilrijkstraat 10, 2650 Antwerpen, Belgium; French Community, Immunization programme, Health department, Office of Birth and Childhood, Chaussée de Charleroi 95, B–1060 Bruxelles, Belgium; Infectious Disease Control and Vaccination, Flemish Agency for Care and Health, Ellipse Building, K. Albert II-laan 35, box 33, B-1030 Brussels, Belgium; French community, School Health Services support, Health department, Office of Birth and Childhood, Chaussée de Charleroi 95, B-1060 Bruxelles, Belgium; Centre for the Evaluation of Vaccination, Vaccine & Infectious Disease Institute, University of Antwerp, Campus 3 Eiken, Universiteitsplein, 1, 2610 Antwerpen, Belgium.

**Keywords:** HPV, vaccination coverage, Belgium, difference, Walloon Region, Flanders, cervical cancer

## Abstract

Long-term results of the HPV vaccination programs in Australia and Scotland have shown a tremendous impact on the reduction of HPV infection rates and precancerous diseases. Both countries started mass vaccination ten years (Australia) and eight years (Scotland) ago and achieved a vaccination coverage of more than 80 %. Within 20 to 30 years a reduction in cervical cancer by more than 75 % is expected. Furthermore, there will be a reduction in other HPV related cancers like vaginal, vulva, perineal, anal and oropharyngeal cancers. In order to be successful, a high vaccination coverage is needed. In Belgium, the vaccination was introduced in 2010 in the Flemish community and in 2011 in the French community. In the first vaccinated cohorts the coverage in Flemish and French Communities was respectively 84% (2010) and 29% (2012-2013).

The latest data suggest that the Flemish Community (Flanders Region) attained a coverage of 91 % while the French Community (Walloon Region) attained a coverage of around 36 %. The regional difference in coverage offers a real-life case. The worst-case scenario could end up with proportionally one half of country having more HPV related cancers than the other half. Currently efforts are performed to increase the coverage rates in both regions and consequently decreasing this difference.

Additionally, the updated recommendations regarding the HPV vaccination by the Belgian NITAG (National Immunization Technical Advisory Group) stated that the HPV vaccination should be gender neutral. This could stimulate the vaccination program and increase the coverage. The coverage rate in Flanders is among the highest in the world and the rate in the French Community is increasing. Efforts should be continued in order to maintain trust and increase the coverage rate.

The current universal HPV (human papillomavirus) vaccination programs in Australia and Scotland have reduced HPV 16 and HPV 18 infections by 89 % and high-grade lesions by 85% seven to ten years after the start (Kavanagh et al., 2017; Garland et al., 2016). At the moment there are no studies regarding the impact on the incidence of cervical cancer. Theoretically it is expected that within 20 – 30 years cervical cancer will be reduced by more than 75% (Tjalma, 2015). In order to be successful a sufficiently high coverage is needed. In Belgium all items for a successful vaccination are available. The advice for the introduction of a HPV vaccination program was made by Belgian NITAG (National Immunization Technical Advisory Group) (also called the permanent vaccination committee of the Superior Health Council) in 2007 and up-dated in 2008 and 2017 (Superior Health Council website, 2018). Prevention in public health, however, is a subnational responsibility and mandate. The decision making for implementation occurs, therefore, at the subnational levels (Flemish Community, French Community). Initially the difference in HPV vaccination coverage between the two communities was estimated at almost fifty-five percent (Flemish Community 84 %; French Community 29 %). The latest Flemish coverage is 91 % and French SHS (School Health Services) coverage study showed a 36.1% coverage (Vermeeren and Goffin, 2018). The latter has to be considered a minimal estimation as a part of the data on immunization administrated by professionals outside the School Health Services (SHS) is lacking. Nevertheless, this means that there is still a 55 percent difference between the Flemish and the French community.

Within one country this offers the opportunity of an experiment of nature regarding the impact of HPV vaccination on cervical cancer rates and other HPV related diseases. There is a huge difference in HPV vaccination coverage in the industrialized world depending on many factors; in particular the way the program is implemented (Hopkins et al., 2013). A survey in the US showed that HPV vaccines have an effectiveness of more than 80%. The effectiveness is defined as the reduction in HPV prevalence of the HPV vaccine types in the vaccinated versus the non- vaccinated population (Markowitz et al., 2013). It needs to be understood that the pre-vaccination HPV prevalence is a key driver of the overall effectiveness of a vaccination program through its influence on the strength of herd protection (Baussano et al., 2018). The pre-vaccination HPV prevalence in Belgian women with normal cytology was 8.4 %, for HPV 16 and 18 this is respectively 2.5 % and 0.9 % (de Sanjosé et al., 2007). The differences in sexual behaviours are also a key factor of the basic reproductive number of HPV infection in different populations. For instance, in populations with gender-equal sexual behaviour a decrease to 1/1000 HPV 16 from prevaccination prevalence of 1% and 8% would require coverages of 37% and 96%, respectively (Baussano et al., 2018).

The HPV vaccination program, with the bivalent and quadrivalent have the potential to reduce cervical cancer by more than 75% (Tjalma, 2015). Based on the HPV type distribution in adult women diagnosed with invasive cervical cancer in Belgium the ninevalent vaccine could raise the number of preventable cervical cancers above 94% (Tjalma et al., 2013; Tjalma et al., 2015).

The reduction figures could be jeopardized by suboptimal level of coverage. There are different factors and issues that can explain the successes and failures of vaccination programs in general, and HPV immunization programs in particular: the cost of the vaccine, the political will, the support of professional organization, the attitude of parents, adolescents and health care workers, the media, just to name a few, seem to play a decisive role (Hopkins et al., 2013; Brabin et al., 2008). The difference in coverage and success of implementation of the universal HPV vaccination program in the different regions of the Federal State of Belgium illustrates the potential role of each of these factors. The general public has accepted the HPV vaccine; however, the acceptance seems to be more anchored in the Flemish-speaking population than in the French- speaking population. In the French Speaking part, you still see more refusal for HPV vaccination than that for other vaccinations (around 10% refuses HPV vaccination). It must be said that not all of them are categorical refusal but more delays (saying that vaccination at 13-14 years is too early and that they will do it later). It shows that in the French speaking part there is still work to be done in order to convince the parents and/or adolescents (Vermeeren and Goffin, 2018).

The HPV vaccines are contracted through a public tender by each of the communities. It also includes a cold chain monitored transport and a direct delivery to the vaccinators. The vaccines are offered free of charge to the target group of young adolescent girls; thus, the cost is not an issue. The coverage of the universal HPV vaccination program however is a success in one half of the country and remains a challenge in the other half.

The HPV vaccines Gardasil® and Cervarix® were introduced in Belgium, respectively in September 2006 and October 2007. They could be bought on prescription in the pharmacies, with different co- payment systems (Lefevre et al., 2011). In Belgium, the National Immunization Technical Advisory Group (NITAG) - Superior Health Council, formulates recommendations on vaccination at National level. Taking the example of the successful universal hepatitis B immunization program through school health services (SHS), general practitioners, paediatricians, and Well-Baby clinics, the NITAG made a first recommendation on May 2nd 2007 for universal HPV vaccination through the SHS or the treating physician for one female age cohort between 10-13 years of age (FitzSimons et al., 2007; Superior Health Council website, 2018). All schools with a recognized learning program are mandatory connected with the SHS in both communities. In September 2010, the Flemish community (6,350,765 inhabitants) did start with an organized HPV vaccination through SHS and treating physicians, with Gardasil® vaccine for girls aged 12 years. The French community (3,435,879 inhabitants) joined one year later, an organized HPV vaccination was offered from September 2011, with Cervarix® vaccine for girls 13-14 years of age. The school- based program is free of charge in both regions.

Depending on the region in Belgium, however, the coverage rate is a success or remains a challenge. In the first vaccinated cohorts (three doses) the coverage in Flemish (2010) and French (2011) communities was respectively 84% (2010) and 29% (2012-2013). The data of 2016 revealed that in the Flemish region the coverage rate for a completed schedule in 16-year-old girls vaccinated in 2012 (at age of 12-13 years) was 91 % (Vandermeulen et al., 2017). These Flemish coverage rates for HPV vaccination are the highest of Europe at the moment. In the middle of 2018 the new HPV coverage report in the French Community was published and this suggested a coverage of 36.1% (Vermeeren and Goffin, 2018). The new data show that the difference in coverages between the communities remains 55%.

Flanders has a “tradition” of adolescent vaccination mostly through the SHS and additionally through treating physicians, with a clearly defined mandate for school doctors for vaccination within the SHS tasks since 2000. The system of SHS is more or less a continuation of the vaccination program of the Well-Baby’s Clinics in Flanders (Kind en Gezin). A reason for this difference is that in the French Community vaccination against HPV through SHS is less well implemented than in Flanders (only 68% of the eligible girls are affiliated to a SHS who propose HPV vaccination and in total 71% of the SHS propose this vaccination) (Vermeeren and Goffin, 2018).

The school-based programs are free of charge and pretty similar in both regions. In case the vaccines are administered by another physician there is a co-payment for the consultations; all vaccinating physicians are informed on HPV vaccination and all vaccinating physicians can order HPV vaccines beforehand, so the vaccines are available “on demand”. In the Flemish region there is a strict approach, population-wide for all girls in the cohort of first year of secondary school. The French SHS sends also an invitation for HPV vaccination (that can be done by their GP or by the SHS if offered) to all girls entering second year of secondary school.

In Flanders a lot of attention was paid to transparent communication. All adolescents, in the Flemish community, entering the first class of secondary school receive a leaflet on vaccination together with the link to a website (Zorg en Gezondheid https://www.zorg-en-gezondheid.be/vaccinatie-tegen-hpv).The website is very informative. In 2018 information about gender neutral vaccination was included. Likewise, efforts have been done to inform the parents in the French speaking part: a leaflet was created in 2011, a new version was update in 2014 (2 doses schedule) and a new version in 2018 (more gender neutral) was created. Additionally, all adolescents receive a leaflet (E-vax, https://www.e-vax.be/VaccHelp/help/pdf/vaccination_13-14_ans.pdf) where the link to an information site on vaccination is given (https://www.vaccination-info.be). On this website further information about vaccination is available. The parents can choose to let their daughter be vaccinated by the SHS, by another physician (paediatrician, gynaecologist or GP), or to refuse the offer.

The accessibility and the organization play an important role in the vaccination coverage. Within the Flemish SHS (CLB) there is a strict organization. The Flemish Scientific Society for Youth Health care (Vlaamse Wetenschappelijke Vereniging voor Jeugdgezondheidszorg (VWVJ)) makes guidelines in order of the Flemish Goverment (www.vwvj.be). These guidelines are available on the website: www.vwvj.be and must be followed by all the Flemish SHS. At the beginning of the school year the parents are informed about the school physician and the Flemish SHS. Parents are asked to sign a letter in which they agree with the preventive care of the school physician and the vaccination program. Each year at the start of the school year a document is also sent to all SHS with the guidelines for the program of vaccination. The difference is that these are only recommendations.

The immunization calendars in SHS (table 1) (Flemish and French) are adapted from the recommendation of the NITAG. The differences in timing are due to practical consideration, especially when the mandatory medical examination of the SHS is realised. There is just a difference of a one year between when it’s done in the Flemish Community and the French speaking Community.

**Table I:** Immunization calendars in the Flemish and French speaking community

Vaccination	Flemish Community	French speaking community
IPV-DTPa	1st year primary school	2nd year primary school (catch-up vaccination for children who didn’t receive the recommended dose at 5-6 years old)
MMR	5th year of primary school	6th year of primary school
HPV	1st year of secondary school (girls only)	1st year (differentiate) and 2st year of secondary school or 13-14-year-old in the specialised school system
dTpa	3rd year of secondary school	4th year of secondary school

In the French community, vaccination is a mandatory mission of SHS since 2001. All SHS are asked to state the pupil’s vaccination status at least 6 times along the pupil school years and offer vaccination in 2nd year primary school (if needed dTpa polio and meningitidis C catching up), 6th year primary school (MMR, and if needed hepatitis B catching up), 2nd year secondary school (HPV), and 4th year secondary school (dTpa). However, for several reasons notably (but not only) financial reasons, the vaccination offer by SHS is not always complete.

Additionally, the role of the media is completely different. In Flanders the local TV and radio stations do not have a negative view on HPV vaccination. The French speaking population in Belgium on the otherhand, is influenced by the French media and it is well known that France is one of the most anti-vaccine countries in the world. Recently this appears to be changing. The trigger for this was the introduction of the mandatory vaccination against 11 sicknesses (2018) in France. Since then multiple articles were published in the media to inform the public in an untestable but scientific way (Le Monde (1,2,3), 2018; Le Figaro (1,2), 2018; Libération (1,2,3), 2018). Furthermore, the official website has been completely reviewed and made more accessible for the public (Official vaccination website France, 2018).

The electronic immunization ordering system linked to a database or register is a major benefit. In Flanders the Well-baby Clinics and SHS work with the electronic immunization ordering system Vaccinnet which is linked to a vaccination database. While in the French Community all the SHS are using an electronic ordering system which is linked to a vaccination register (e-vax.be). At the moment efforts are done to convince all professionals in the French community to use this system. For all vaccines ordered in the system it is mandatory to register the given vaccinations. First of all, the vaccinators can access this database to see if someone is vaccinated or not. And secondly the HPV vaccination records in this database will allow in the future the evaluation of the impact of the HPV vaccination on cervical cancer in Belgium, if data- linking between databases is possible. Furthermore, if data from both databases are linked to data from a screening and pathology database one can find out which types are responsible for cervical cancer in the vaccinated cohort, if ever, and compare with the non-vaccinated cohort. Therefore you need a common identifier which exists in Belgium - everybody has a personal national registry number. The lack of data on previous vaccination is one of the reasons why vaccination is postponed or not done. For the SHS this is a real problem as they don’t know if the individual should be vaccinated or not and it takes a lot of time and effort to find the answer (numerous phone calls to the parents) if any. The impact of a vaccination program depends on the coverage rate. Even within a country with the resources for mass vaccination program uptake seems to vary. The success of a vaccination program depends on the support of all stakeholders: the public acceptance, the political will, and the support of the public and healthcare professionals are essential. General barriers are 1) vaccine costs and reimbursement including insurance, (2) storage and/ or monitoring of vaccines, (3) vaccine knowledge, (4) provider and patient attitudes toward vaccination, (5) provider and patient concerns about vaccine safety, (6) obtaining appropriate consent from adolescents’ parents or from themselves and (7) missed opportunities for adolescent immunization (Hopkins et al., 2013).

In Belgium most of these items were addressed successfully yet there was a huge difference in coverage rate between the two regions. Missed opportunities for adolescent immunization is still an issue in the French part of the country mostly due to the fact that not all SHS offer HPV vaccination.

As soon as we will link data from disease and immunization database, we will be able to measure the impact of HPV vaccination on cervical cancer rates and other HPV related diseases in both parts of the country. The result difference will reflect the impact of the coverage rate.

In the recent scientific advisory report on HPV vaccination, the Superior Health Council of Belgium recommends gender neutral prophylactic HPV vaccination in a two dose schedule for 9 to 14 years and in a three dose schedule for 15 – 26 years old (Superior Health Council website, 2018). If this recommendation is followed and a high coverage is achieved, then the HPV related precancers and cancer will be strongly reduced if not almost eradicated in Belgium. The gender neutral could be a way to stimulate the vaccination program and increase the coverage

## Conclusion

The availability of a HPV vaccine in a country is not a guarantee of success, not even when it is offered free of charge. The situation in both communities in Belgium illustrates the role different factors may play in different ways: differences in awareness, spill over effects of the media in neighbouring country, different penetration of vaccination by SHS in each part of the country. Understanding the role of these factors and tailoring the implementation of the HPV programme to the needs of each community is key, in addition to efforts to raise knowledge and awareness among the respective health care providers and parents in each part of the country.
